# The *Bursaphelenchus xylophilus* Effector BxNMP1 Targets PtTLP-L2 to Mediate PtGLU Promoting Parasitism and Virulence in *Pinus thunbergii*

**DOI:** 10.3390/ijms25137452

**Published:** 2024-07-07

**Authors:** Dan Yang, Lin Rui, Yi-Jun Qiu, Tong-Yue Wen, Jian-Ren Ye, Xiao-Qin Wu

**Affiliations:** 1 Co-Innovation Center for Sustainable Forestry in Southern China, College of Forestry, Nanjing Forestry University, Nanjing 210037, China; yangdan@njfu.edu.cn (D.Y.); ruilin0305@njfu.edu.cn (L.R.); qiuyijun722@126.com (Y.-J.Q.); tywen940822@126.com (T.-Y.W.); 2 Jiangsu Key Laboratory for Prevention and Management of Invasive Species, Nanjing Forestry University, Nanjing 210037, China

**Keywords:** *Bursaphelenchus xylophilus*, effector, thaumatin-like protein, β-1,3-glucanase, salicylic acid, *Pinus thunbergii*

## Abstract

*Pinus* is an important economic tree species, but pine wilt disease (PWD) seriously threatens the survival of pine trees. PWD caused by *Bursaphelenchus xylophilus* is a major quarantine disease worldwide that causes significant economic losses. However, more information about its molecular pathogenesis is needed, resulting in a lack of effective prevention and treatment measures. In recent years, effectors have become a hot topic in exploring the molecular pathogenic mechanism of pathogens. Here, we identified a specific effector, BxNMP1, from *B. xylophilus*. In situ hybridization experiments revealed that *BxNMP1* was specifically expressed in dorsal gland cells and intestinal cells, and RT–qPCR experiments revealed that *BxNMP1* was upregulated in the early stage of infection. The sequence of *BxNMP1* was different in the avirulent strain, and when *BxNMP1*-silenced *B. xylophilus* was inoculated into *P. thunbergii* seedlings, the disease severity significantly decreased. We demonstrated that BxNMP1 interacted with the thaumatin-like protein PtTLP-L2 in *P. thunbergii*. Additionally, we found that the β-1,3-glucanase PtGLU interacted with PtTLP-L2. Therefore, we hypothesized that BxNMP1 might indirectly interact with PtGLU through PtTLP-L2 as an intermediate mediator. Both targets can respond to infection, and PtTLP-L2 can enhance the resistance of pine trees. Moreover, we detected increased salicylic acid contents in *P. thunbergii* seedlings inoculated with *B. xylophilus* when *BxNMP1* was silenced or when the PtTLP-L2 recombinant protein was added. In summary, we identified a key virulence effector of PWNs, BxNMP1. It positively regulates the pathogenicity of *B. xylophilus* and interacts directly with PtTLP-L2 and indirectly with PtGLU. It also inhibits the expression of two targets and the host salicylic acid pathway. This study provides theoretical guidance and a practical basis for controlling PWD and breeding for disease resistance.

## 1. Introduction

*Pinus* is an important tree species in the global ecosystem. It has economic, medicinal, and ornamental value. However, pine wilt disease (PWD) caused by the pine wood nematode (PWN) *Bursaphelenchus xylophilus* is the most devastating pine tree disease. It leads to substantial economic losses in European and East Asian countries, particularly in China and Japan [1]. The feeding strategies of PWNs include phytophagous and mycophagous stages. At the phytophagous stage, PWNs migrate to host pine trees and feed on parenchyma cells. When PWNs multiply in large numbers in pine trees, the water delivery system of the infected pine is destroyed, leading to wilting and eventual death [1]. Once PWD occurs and becomes difficult to control, its pathogenesis involves complex interactions between pathogenic nematodes, insect vectors, and host pines [2]. Therefore, the molecular pathogenic mechanism of PWD is a difficult research topic, but preventing and controlling this disease is also a hot topic.

To combat the invasion of foreign pathogens, plants have developed a plant immune system. The first layer of the plant immune system involves the recognition of pathogen-associated molecular patterns (PAMPs) by pattern recognition receptors (PRRs) in plants, a process known as PAMP-triggered immunity (PTI) [3]. To overcome the plant immune response (PTI), pathogens secrete effectors into host cells to interfere with the plant’s immune system and promote self-infection [4]. However, in response to attack by pathogenic effectors, plants develop new immune responses, namely, effector-triggered immunity (ETI) [3,4]. Numerous plant-parasitic nematode (PPN) effectors have been demonstrated to suppress plant immunity. They secrete effector proteins into plants via the stylet in their mouthparts [5]. These effectors play vital roles in the infection stages. For example, the effectors of *Globodera pallida* were shown to inhibit nucleotide binding/leucine-rich-repeat (NLR) protein-induced defense responses [6,7]. In recent years, an increasing number of PWN effectors have been predicted and identified through transcriptomics [8,9,10]. BxSapB1 is the first reported PWN effector that can trigger cell necrosis in *Nicotiana benthamiana* and regulate PWN virulence [11]. Subsequently, BxSapB2 and BxSapB3, which have the same domain, were also reported [12,13]. BxSCD1 is the first reported PWN effector that can suppress host immune responses; it inhibits cell death in *N. benthamiana* and *P. thunbergii* and interacts with the ethylene synthase PtACO1 in *P. thunbergii* [14]. Subsequently, PWN effectors with FAR, ML, and Lip domains were reported. They inhibit host immunity by interacting with different host targets and exerting various functions [15,16,17]. In general, effector proteins containing specific domains are usually enzymes. They are easily degraded during infection. Additionally, to successfully infect the host, the pathogen often needs to secrete a large number of effectors. Thus, there must be effectors that play key roles in virulence. Moreover, current research on PWN effectors often stops at the identification of host targets without delving deeper into the functions of these targets or uncovering the host pathways associated with target proteins. The aim of this study was to identify a key virulence factor of PWNs and reveal the complete interaction pathway between the effector and the host.

Plant thaumatin-like protein (TLP) and β-1,3-glucanase are pathogenesis-related proteins (PRs). TLP belongs to the PR5 family, and β-1,3-glucanase belongs to the PR2 family [18]. The TLP family is a highly complex family of proteins closely associated with plant resistance and strongly linked to transgenic-resistant plants [19]. For example, when the *AdTLP* gene is overexpressed in *Arachis diogoi*, resistance to the pathogenic fungus *Rhizoctonia solani* is significantly enhanced [20]. Transgenic *Pe-TLP* poplars exhibit enhanced resistance to spot disease [21]. Additionally, several studies have shown that pathogen effectors interact with plant TLPs to suppress plant immunity. For example, the *Trichoderma virens* Alt a 1 protein may target TLP to suppress plant defense [22]. The *Puccinia triticina* effector Pt_21 interacts with the wheat thaumatin-like protein TaTLP1 to inhibit its antifungal activity and suppress wheat apoplastic immunity [23]. β-1,3-glucanase also plays an important role in plant growth, development, and resistance to pathogens [24]. On the one hand, β-1,3-glucanase primarily participates in plant defense by breaking down the cell wall of pathogens, often working synergistically with chitinase to degrade the fungal cell wall [25]. On the other hand, β-1,3-glucanase and some PR proteins can act as elicitors. These signals are recognized by plant surveillance systems and are transduced through signaling cascades, leading to the initiation of a wide range of localized and systemic defense responses [26]. Based on their importance, many scholars have co-overexpressed these PR proteins in plants to enhance plant resistance [26]. Although the significance of these PR proteins in plant disease resistance is known, the specific links between them remain poorly understood.

Phytohormones are crucial endogenous factors that mediate plant biotic and abiotic stress, serve as integration centers for plants in response to environmental stress, and play a vital role in plant defense mechanisms. Fine regulation of the stress response is achieved through complex signaling networks and intricate cross-talk between different hormone signaling pathways [27]. Salicylic acid (SA) is typically involved in defense responses against living vegetative and semiliving vegetative pathogens or pests [28]. Previous studies revealed that there are two main pathways for salicylic acid (SA) biosynthesis in plants: the isochorismate synthase (ICS) pathway and the phenylalanine ammonia lyase (PAL) pathway [29,30]. In the ICS pathway, glutamate is involved in the synthesis of plant SA [31]. However, the specific changes in the synthesis of SA in the PAL pathway are not known [32]. A common strategy used by pathogens to bypass the plant’s hormonal defense system is to secrete effectors. The effector Cmu1 secreted by *Ustilago maydis* encodes a chorismate mutase that converts chorismate, a precursor of SA, to prephenate. In plants infected with the Cmu1 deletion mutant, the SA levels increased 10-fold compared to those in plants infected with the wild-type strain [33]. SA is also required for resistance to PPNs. For example, the *Heterodera schachtii* effector acts on Arabidopsis SA signal transduction [34,35], and the beet cyst nematode effector HsSNARE1 interacts with AtSNAP2 and AtPR1 to affect the SA pathway in *Arabidopsis*, promoting nematode infection [36]. This suggests that nematodes have also evolved the ability to interfere with the host SA pathway. However, little is known about how PWN effectors intervene in the systemic acquired resistance (SA) pathway.

The identification of PWN effectors has significantly enhanced the understanding of the molecular pathogenesis of PWD. Pathogens often need to secrete a large number of effectors into host plants to achieve successful invasion. Therefore, there must be effectors that are specific to PWNs and have key effects on the virulence of PWN. The aim of this study was to explore the function of a key virulence effector specific to PWN. Based on the specific mechanism of the interaction between PWNs and host pine trees, this research will contribute to the prevention and control of PWD and the breeding of pine trees with disease resistance.

## 2. Results

### 2.1. Identification of the B. xylophilus Candidate Effector BxNMP1

According to the transcriptome data [9], we identified a specific effector gene (BXY_1088700) with a signal peptide but without known domains that was upregulated in the transcriptome. Natural populations of PWNs exhibit varying levels of virulence. To explore the sequence polymorphisms of *BxNMP1* in different PWN isolates, we sequenced the *BxNMP1* (nematode manipulator of pathogenesis-related protein 1) coding region from nine PWN strains with varying virulence and from various sources: AMA3 (highly virulent strain), LN16 (highly virulent strain), ZJ07 (highly virulent strain), CQ09 (moderately virulent strain), GX11 (moderately virulent strain), YW4 (weakly virulent strain), JX06 (weakly virulent strain), OKD (weakly virulent strain), and GD15 (weakly virulent strain) [37]. The results showed that the gene sequence had a five-base pair mutation in the GD15 isolate (weakly virulent strain) that caused a mutation at amino acid 8 (Figure S1a,b). Thus, it was speculated that the effector may be related to PWN virulence. To further predict the function of BXY_1088700, we constructed a protein model and found that it comprises two α-helices and three antiparallel β-strands (Figure S1c).

To conduct a preliminary exploration of the function of candidate effectors in the host, *A. tumefaciens* carrying BXY 1088700 (without a signal peptide) was injected into *N. benthamiana* leaves 16 h prior, followed by *A. tumefaciens* carrying *BxCDP1* into *N. benthamiana*. We found that BxCDP1-triggered cell death was suppressed at 5 days postinoculation (dpi) (Figure S1d). Electrolyte leakage in *N. benthamiana* triggered by BxCDP1 was significantly reduced in the presence of the candidate effector (Figure S1e). Western blot analysis confirmed the expression of proteins (BXY 1088700-RFP, BxCDP1, and red fluorescent protein [RFP]) in *N. benthamiana* leaves (Figure S1f). These results show that the candidate effectors function in plant cells and suppress cell death in *N. benthamiana*. Therefore, we chose this protein for further study and named it BxNMP1 based on its functionality.

### 2.2. BxNMP1 Is Upregulated during Infection and Is Localized to Dorsal Gland Cells and the Intestine

**Figure 1:**
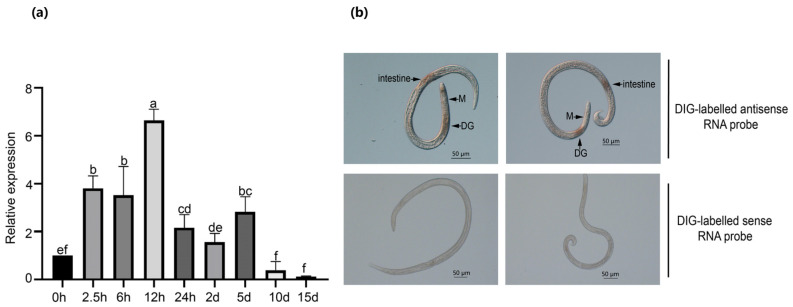
Expression pattern of *BxNMP1*. (**a**) The relative expression level of *BxNMP1* at nine time points after *Bursaphelenchus* xylophilus infection was determined using reverse transcription-quantitative PCR (RT–qPCR) analysis. Nematodes were collected from entire *Pinus thunbergii* seedlings inoculated with *B. xylophilus* (isolating AMA3) at various time points. The relative expression level of *BxNMP1* was calculated using the comparative threshold method. The RT–qPCR values were normalized to the transcript level of *Actin*. The values represent the means ± standard deviations of three independent biological samples. Different letters indicate statistically significant differences according to Duncan’s multiple range test (*p* < 0.05). (**b**) Localization of BxNMP1 in the dorsal glands (DGs) and intestine via in situ hybridization. DIG: digoxigenin; M: median bulb. Scale bars = 50 µm.

Changes in the expression of effectors during different stages of pathogen infection are often related to their functions [38]. RT–qPCR was utilized to measure the expression levels of *BxNMP1* at nine infection time points using the highly virulent strain AMA3. The expression of *BxNMP1* continued to increase for 5 days before PWN infection, with the highest expression occurring at 12 h. (Figure 1a). We used in situ hybridization assays to investigate the location of *BxNMP1* expression in PWNs. The results showed that the red location mark was specifically expressed in the dorsal gland cells and intestines of PWNs, but no signal was detected when a sense RNA probe was used (Figure 1b). Collectively, the above results indicate that *BxNMP1* is secreted by the dorsal glands and intestines of PWNs and plays a role in the early stage of infection.

### 2.3. BxNMP1 Contributes to B. xylophilus Pathogenicity

**Figure 2:**
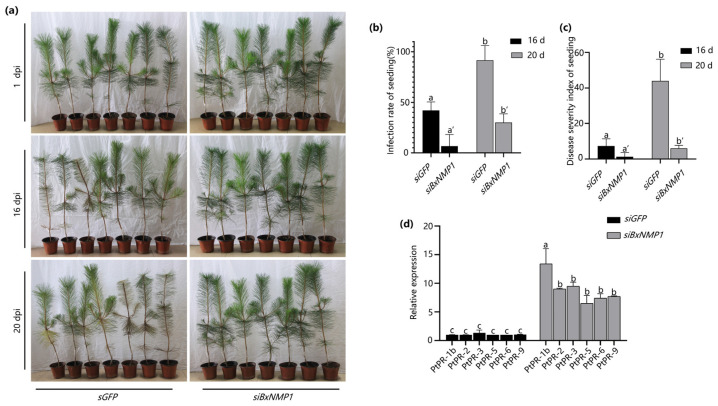
BxNMP1 contributes to the virulence of *B. xylophilus*. (**a**) Inoculation assay of *Pinus thunbergii* seedlings. The degree of morbidity of the *P. thunbergii* seedlings varied according to the color of the needle leaves. At 16 days postinoculation (dpi), four *P. thunbergii* seedlings inoculated with *siGFP*-treated *B. xylophilus* turned yellow, but none of the *P. thunbergii* seedlings inoculated with *siBxNMP1*-treated *B. xylophilus* turned yellow. At 20 dpi, all the *P. thunbergii* seedlings inoculated with siGFP-treated *B. xylophilus* turned yellow or brown, and one *P. thunbergii* seedling inoculated with *siBxNMP1*-treated *B. xylophilus* turned yellow. (**b**) The infection rates of *P. thunbergii* seedlings under different treatments. (**c**) The disease severity index of *P. thunbergii* seedlings under various treatments. (**d**) The relative transcript levels of pathogenesis-related genes in *P. thunbergii*-infected *siBxNMP1*-treated nematodes were upregulated compared with those in control nematodes. Stems approximately 1 cm in length were selected for RNA extraction at 12 h postinoculation. The data are presented as the means ± standard deviations (SD) from three biological replicates. Different letters indicate significant differences (*p* < 0.05).

To assess the contribution of BxNMP1 to the pathogenicity of PWNs, we used RNA interference (RNAi) to silence *BxNMP1* expression in the AMA3 isolate. We inoculated *P. thunbergii* seedlings with PWNs by soaking them in an siRNA solution for 48 h. RT–qPCR confirmed that *BxNMP1* was successfully silenced, and the transcript levels decreased by approximately 50% (Figure S2a). *P. thunbergii* plants inoculated with PWNs treated with *the siGFP* control had significantly yellow needles at 16 dpi, and nearly half of the *P. thunbergii* seedlings were diseased. However, *P. thunbergii* seedlings inoculated with si*BxNMP1*-treated nematodes did not show any symptoms at 16 dpi, and only slight symptoms appeared at 20 dpi (Figure 2a,b). In addition, the disease severity index also demonstrated that silencing *BxNMP1* could delay the onset of the disease (Figure 2c). The *Botrytis cinerea* mycelium on the PDA plates used for culturing the PWNs was observed, and the number of PWNs on the *B. cinerea* plates and on the *P. thunbergii* seedlings was counted. The results showed that *siBxNMP1*-treated PWNs exhibited a significant delay in reproduction in *P. thunbergii* seedlings, while there was no difference in reproductive or feeding rate on *B. cinerea* plates (Figure S2b–d). The data suggest that BxNMP1 is a key virulence factor in PWN infection stages and contributes to fecundity when *B. xylophilus* infects *P. thunbergii*. We also measured the change in the PR gene in pine trees at 12 h postinoculation and found that the expression of most of the PR genes significantly increased in *P. thunbergii* seedlings inoculated with *siBxNMP1*-treated PWNs compared with those inoculated with *siGFP*-treated PWNs, especially *PtPR-1b* (Figure 2d). These results imply that BxNMP1 plays a significant role in suppressing immune responses in *P. thunbergii*.

### 2.4. BxNMP1 Interacts with the Host Plant Thaumatin-like Protein L2

**Figure 3:**
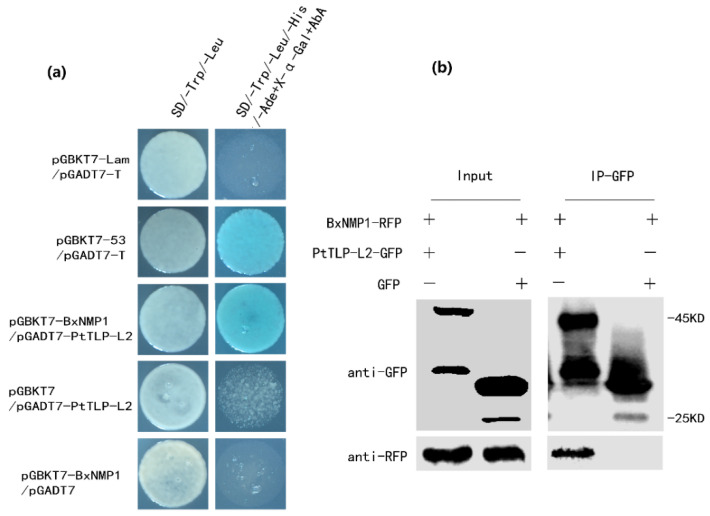
BxNMP1 interacts with PtTLP-L2 in *Pinus thunbergii*. (**a**) BxNMP1 interacts with PtTLP-L2 in yeast. The Y2H Gold yeast strain cotransformed with BD-BxNMP1 and AD-PtTLP-L2 was cultured on SD/−Trp/−Leu media and then on SD/−Trp/−Leu/−His/−Ade+X−α−Gal+AbA selective media. The images were captured using a four-fold magnification of microscope. (**b**) BxNMP1 interacts with PtTLP-L2 in vivo. Coimmunoprecipitation (Co-IP) was performed on extracts of *Nicotiana benthamiana* leaves expressing both BxNMP1-RFP and PtTLP-L2-GFP. Green fluorescent protein (GFP) was detected via Western blot using anti-GFP antibodies. Red fluorescent protein (RFP) was detected via Western blot using anti-RFP antibodies. The immune complexes were pulled down using anti-GFP agarose beads.

These results suggest that BxNMP1 plays an important role in the interaction between *B. xylophilus* and *P. thunbergii*. To explore the targets of BxNMP1 in *P. thunbergii*, we screened the potential targets of BxNMP1 in *P. thunbergii* using a yeast two-hybrid screening assay (the potential targets are listed in Table S2). We identified one major target, thaumatin-like protein-L2 (TLP-L2), which was captured twelve times, whereas other candidates, such as β-1,3-glucanase, were captured fewer times. Subsequently, we confirmed the interaction between full-length BxNMP1 and PtTLP-L2 in yeast (Figure 3a). Co-IP experiments further confirmed this interaction (Figure 3b). The above results indicate that BxNMP1 specifically interacts with PtTLP-L2 in *P. thunbergii.*

### 2.5. PtTLP-L2 Interacts with β-1,3-Glucanase in P. thunbergii

**Figure 4:**
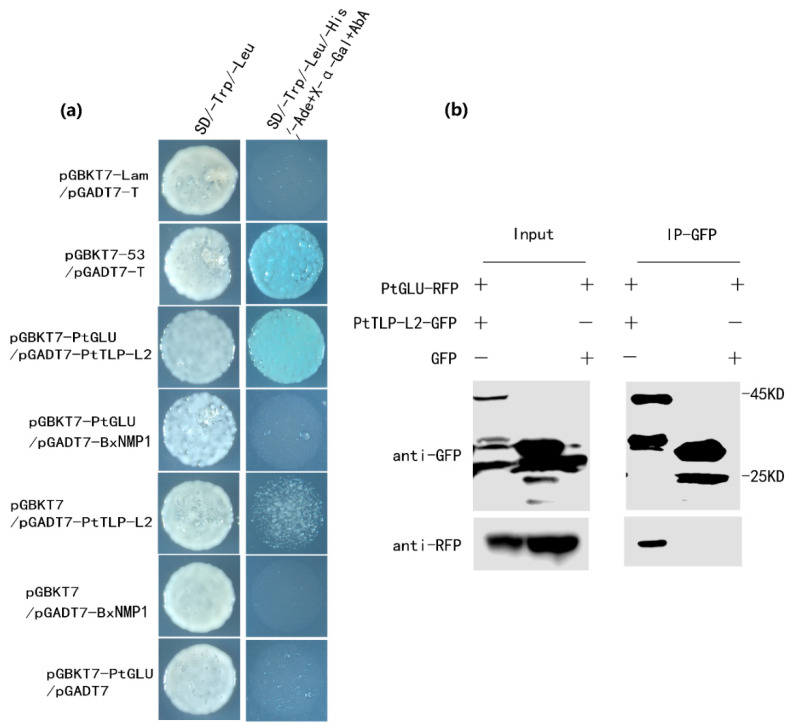
PtGLU interacts with *Pinus thunbergii* TLP-L2 proteins. (**a**) PtGLU interacts with Pt TLP-L2 in yeast. The Y2H Gold yeast strain cocarrying BD-PtGlu and AD-Pt-TLP-L2 was grown on SD/−Trp/−Leu and the selective medium SD/−Trp/−Leu/−His/−Ade+X−α−Gal+AbA. The images were captured using a four-fold magnification of microscope. (**b**) PtGLU interacts with PtTLP-L2 in vivo. Coimmunoprecipitation (Co-IP) was performed on extracts of *Nicotiana benthamiana* leaves expressing both PtGLU-RFP and PtTLP-L2-GFP. Green fluorescent protein (GFP) was detected via Western blot using anti-GFP antibodies. Red fluorescent protein (RFP) was detected via Western blot using anti-RFP antibodies. The immune complexes were pulled down using anti-GFP agarose beads.

Many studies have reported that TLPs have glucanase activity [39,40]. Moreover, we found that β-1,3 glucanase (GLU) was also a potential target of BxNMP1, but the full-length sequences of BxNMP1 and PtGLU cannot interact in yeast (Figure 4a). Therefore, we speculated that BxNMP1 can interact with PtGLU through PtTLP-L2 as an intermediary. We further explored whether PtGlu and PtTLP-L2 could interact with each other. We acquired the entire GLU coding sequence from *P. thunbergii* using a specific primer designed for *Picea asperata* [41]. The Y2H assay demonstrated that PtGLU was able to interact with PtTLP-L2 but not with the effector BxNMP1 (Figure 4a). Subsequently, we further demonstrated the interaction between PtGLU and PtTLP-L2 using CoIP experiments (Figure 4b).

### 2.6. BxNMP1, PtTLP-L2, and PtGLU Colocalize in the Nucleus and Cytoplasm in N. benthamiana

**Figure 5:**
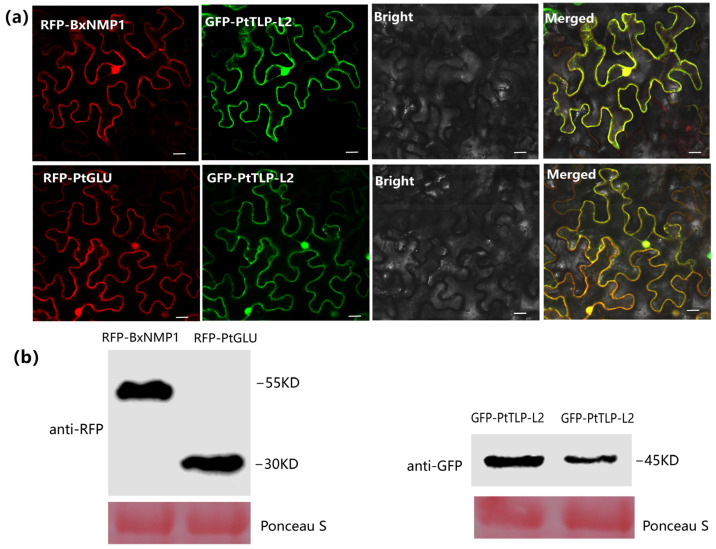
PtTLP-L2 colocalizes with BxNMP1 and PtGLU in the nucleus and cytoplasm. Proteins were expressed in *Nicotiana benthamiana* via agroinfiltration. (**a**) Confocal microscopy imaging of *N. benthamiana* leaves transiently expressing green fluorescent protein (GFP)-tagged PtTLP-L2, red fluorescent protein (RFP)-tagged BxNMP1 and PtGLU, showing that PtTLP-L2 colocalizes with BxNMP1 and PtGLU in both the nucleus and the cytoplasm. Pictures taken 48 h postinfiltration show cells cotransformed with PtTLP-L2 (green channel; **middle left panel**) and BxNMP1 and PtGLU (red channel; **left panel**). Bright field images (**middle right panel**) and the overlay (**right panel**) are also shown. Scale bars, 10 μm. (**b**) Expression of GFP-PtTLP-L2 together with RFP-BxNMP1 and RFP-PtGLU was confirmed via Western blotting using anti-GFP and anti-RFP antibodies. Protein loading is indicated with Ponceau S staining of RuBisCO.

Understanding the localization of proteins in plant cells is crucial for understanding protein function, gene regulation, and protein–protein interactions [42]. To determine the subcellular localization of BxNMP1 and its targets in plant cells, we examined the colocalization of these proteins. We constructed PtTLP-L2 with green fluorescent protein (GFP). BxNMP1 and PtGLU are connected to red fluorescent protein (RFP) through homologous recombination. Both red fluorescence and green fluorescence were detected in the cytoplasm and nucleus of *N. benthamiana* (Figure 5a). The expression of these proteins was confirmed with Western blotting (Figure 5b). These findings further confirm the possibility of direct or indirect interactions among the three proteins.

### 2.7. PtTLP-L2 Enhances the Resistance of P. thunbergii

**Figure 6:**
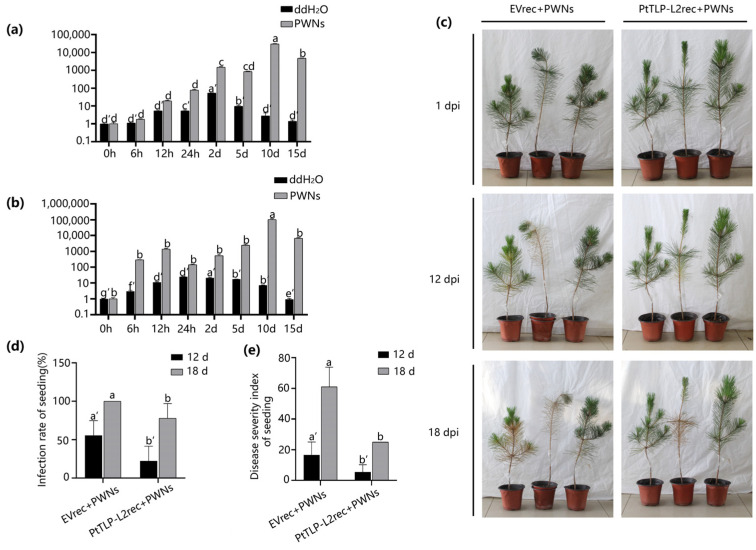
Expression pattern of *PtTLP-L2* and *PtGLU* in infected *Pinus thunbergii,* and PtTLP-L2 can enhance the resistance of *P. thunbergii*. (**a**) The expression of *PtTLP-L2* in infected *Pinus thunbergii* at different time points. (**b**) The expression of *PtGLU* in infected *P. thunbergii* at different time points. In total, 2000 PWNs were inoculated into 3-year-old *P. thunbergii* seedlings. Stems approximately 1 cm in length were collected to analyze the relative expression of *PtTLP-L2* and *PtGLU*. (**c**) The symptoms of *P. thunbergii* at 12 and 18 days postinoculation (dpi) with *Bursaphelenchus xylophilus* and two different purified recombinant proteins (EVrec: pET32a and PtTLP-L2rec). (**d**,**e**) The infection rate and disease severity index of *P. thunbergii* seedlings were calculated at 12 and 18 dpi. Three independent experiments were performed, and at least 3 individual *P. thunbergii* seedlings were used for each treatment. The data are presented as the means ± standard deviations (SD) from three experiments. Different letters indicate significant differences (*p <* 0.05).

The experiment demonstrated that BxNMP1 directly interacts with PtTLP-L2 and indirectly interacts with PtGLU through PtTLP-L2 as an intermediary. Therefore, we wanted to further explore how *PtTLP-L2* and *PtGLU* respond to PWN infection. We used RT–qPCR technology to detect the expression levels of *PtTLP-L2* and *PtGLU* at different time points in *P. thunbergii* seedlings infected with PWNs. To reduce the effects of wounds on target gene expression levels, another group of seedlings was inoculated with sterile water at similar inoculation sites. The expression levels of *PtTLP-L2* peaked at 10 dpi when the pine needles began to show yellow symptoms (Figure S3), increasing by approximately 30,000-fold. This differed significantly from the control group that was inoculated with sterile water (Figure 6a). The expression pattern of *PtGLU* was broadly consistent with the trend of *PtTLP-L2*. The expression level reached a small peak at 12 h postinoculation, and its highest peak appeared at 10 dpi (Figure 6b). The results demonstrated that *PtTLP-L2* and *PtGLU* in pine trees are highly upregulated in response to nematode infection.

The host target was indeed able to respond to PWN infection, and its expression was strongly upregulated. However, why did strong upregulation fail to stop the pine tree from dying? We speculate that the reason is that PtTLP-L2 was strongly upregulated during the late stage of infection, when the PWNs were dominant between the interactions of PWNs and pine trees. We used the method of inoculating the PtTLP-L2 recombinant protein (PtTLP-L2rec) to ensure that the host target was highly expressed at the beginning of infection. We obtained purified PTTLP-L2rec in vitro. The empty vector (EV) pET32a was used as a negative control. Subsequently, PtTLP-L2rec and EVrec were analysed using SDS–PAGE and Western blot analysis to confirm successful purification (Figure S4). We inoculated 1500 PWNs into *P. thunbergii* seedlings and subsequently quantified 200 µg of PtTLP-L2rec or EVrec. More than half of the *P. thunbergii* seedlings inoculated with EVrec began to show obvious symptoms, while only one *P. thunbergii* seedling inoculated with PtTLP-L2rec showed slight symptoms at 12 dpi. One *P. thunbergii* seedling inoculated with EVrec died, and the others also developed severe symptoms. In contrast, only one *P. thunbergii* seedling inoculated with PtTLP-L2rec showed obvious symptoms at 18 dpi (Figure 6c). The incidence rate and disease severity index also demonstrated that inoculation with PtTLP-L2rec reduced the severity of PWD (Figure 6d,e). The results showed that overexpression of PtTLP-L2 at the initial stage of infection could enhance the resistance of *P. thunbergii* and reduce the severity of the disease.

### 2.8. BxNMP1 Inhibits the Expression of Targets and Suppresses the Salicylic Acid Pathway in P. thunbergii

**Figure 7:**
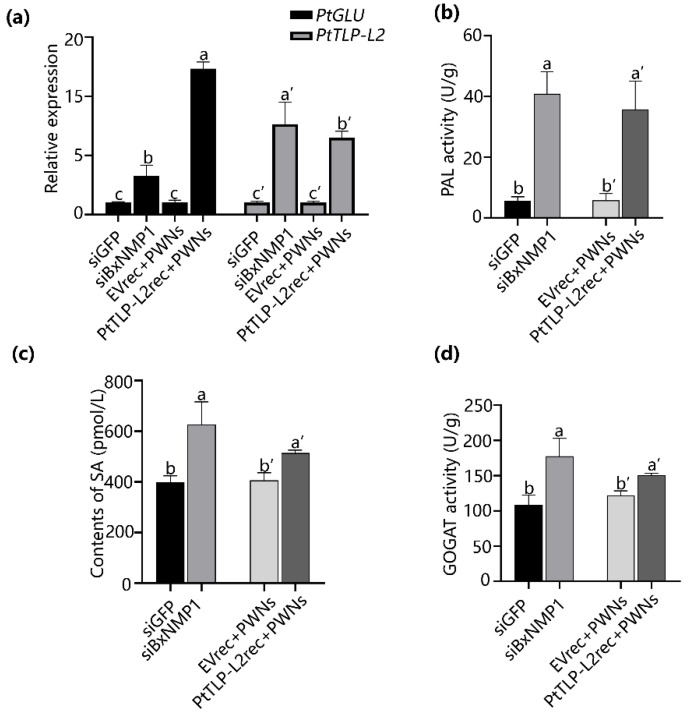
Changes in the activity of the salicylic acid pathway in *Pinus thunbergii* under different treatments. In total, 2000 *siBxNMP1*-treated nematodes, *siGFP*-treated nematodes, a total of 200 µg of purified PtTLP-L2 recombinant protein (PtTLP-L2rec) or EV recombinant protein (EVrec), and untreated PWNs were inoculated into 3-year-old *P. thunbergii* seedlings. Stems approximately 1 cm in length were selected for measurement of enzyme activity or for extraction of RNA at 12 h postinoculation. (**a**) Relative expression of *PtTLP-L2* and *PtGlu* in *P. thunbergii* seedlings under four treatments. (**b**–**d**) Contents of salicylic acid, PAL, and GOGAT activity in *P. thunbergii* seedlings under four treatments. The values represent the means ± SDs of three independent biological samples. Different letters on top of the bars indicate statistically significant differences (*p* < 0.05, *t* test), as measured with Duncan’s multiple range test.

To further elucidate the interaction between the effector BxNMP1 and its targets, we inoculated *P. thunbergii* seedlings with *siBxNMP1*-treated PWNs, *siGFP*-treated PWNs, purified PtTLP-L2 protein, or EV protein. This study was performed to investigate the impact of BxNMP1 on the host immune system at 12 h postinoculation. The expression of *PtTLP-L2* and *PtGLU* was also significantly upregulated in the plants inoculated with *siBxNMP1*-treated PWNs or PtTLP-L2rec. In addition, the upregulation of *PtGLU* in seedlings inoculated with PtTLP-L2rec was greater than that in seedlings inoculated with *siBxNMP1*-treated PWNs (Figure 7a).

*PR1-b* is a marker gene of the systemic acquired resistance (SA) pathway, and it is significantly upregulated in pine trees inoculated with PWNs in which *BxNMP1* is silenced (Figure 2d). In addition, PtTLP-L2 belongs to the PR5 family, and PtGLU belongs to the PR2 family. Both are SA response genes [43,44]. Thus, we analyzed the contents of SA as well as the activity of key enzymes involved in the isochorismate synthase (ICS) synthesis pathway of SA in four treated pine seedlings. The results showed that the levels of SA, PAL, and GOGAT activities in *P. thunbergii* seedlings increased when they were inoculated with *siBxNMP1*-treated PWNs or purified PtTLP-L2 proteins (Figure 7b–d). PtTLP-L2 can activate the defense response in pine trees, whereas BxNMP1 can inhibit the expression of *PtTLP-L2* and *PTGLU* and inhibit the SA pathway in pine trees.

## 3. Discussion

PWD constitutes a severe forest disease, causing the death of numerous pine trees every year [45]. Studies have shown that PWD may be caused by the strong immunity of the host itself after pathogen invasion, which leads to the death of pine trees. Futai and Myers postulated that the spread of PWNs through a tree causes a series of hypersensitive responses that eventually lead to the death of susceptible pines [1,45]. Hirao compared gene expression patterns between resistant and susceptible pine trees after PWN infection and found that the expression levels of PR genes (e.g., PR-1b, PR-2, PR-3, PR-4, PR-5, and PR-6) were much greater in susceptible trees inoculated with virulent PWNs than in resistant trees at all time points [43]. Yamaguchi showed that all pine plants in which the number of PWNs and the expression levels of PR genes increased after PWN inoculation exhibited external symptoms [46]. However, these studies were based on the physiological and biochemical symptoms of pine trees and did not delve into the interaction between PWNs and pine trees. In recent years, effectors have become a focal point for elucidating the pathogenic mechanism of pathogens. Plant-parasitic nematode (PPN) effectors, primarily originating from three highly specialized gland cells within the nematode, are secreted proteins that manipulate various host cell processes, including defense responses, to achieve successful parasitism [6,7,14,35]. At present, research on PPN effectors has focused mainly on root-knot nematodes and cyst nematodes. The study of the effectors of PWNs started late. We screened effectors using transcriptome data from the early stage of PWN infection [9]. BLAST analysis of the NCBI database indicated that the BxNMP1 sequence is specific to *B. xylophilus* and does not contain a known domain. In general, proteins that do not contain known domains are not enzymes and are not easily degraded, and they often play an important role in the process of infection. We analyzed the sequences of *BxNMP1* from different sources and virulent PWN strains. The sequence of *BxNMP1* was mutated in the weakly virulent strain GD15, resulting in a change in amino acid 8 (Figure S1a,b). Sequence analysis of *BxNMP1* revealed that *BxNMP1* is specific to PWNs and is not a known enzyme. BxNMP1 may play an important role in the infection process. The difference in sequences of the avirulent strains also suggested a correlation with virulence.

The gene *BxNMP1* was highly expressed within 5 days after PWN infection of the pine trees. Unlike several previously studied effectors of PWNs that were slowly upregulated in the early infection stages and then peaked at 12 h [14,16], *BxNMP1* was highly upregulated at 2.5 h, peaked at 12 h, and remained highly expressed until 5 d (Figure 1a). Based on multiple transcriptome data, Hu reported that the host cell wall may be degraded by hydrolases and lyases secreted by PWNs and that PWN effectors play a key role in ROS removal through catalysis and redox reactions during 6–12 h of infection [10]. Therefore, BxNMP1 may be involved in overcoming host defense at an earlier stage. In situ hybridization experiments revealed that *BxNMP1* was specifically expressed in the dorsal glands and intestines of PWNs (Figure 1b). A growing body of research suggests that dorsal gland cells gradually expand and become more active during the parasitic phase of nematodes [47]. Therefore, the effectors located in dorsal gland cells are thought to be involved in establishing feeding sites and promoting parasitism. These results suggest that BxNMP1 is secreted into host plant cells through the dorsal glands and intestine of PWNs and plays a role in the parasitic stage.

We silenced *BxNMP1* in PWNs and subsequently inoculated the silenced PWNs into *P. thunbergii* seedlings. In the pine trees inoculated with *siBxNMP1*-treated PWNs, only one *P. thunbergii* seedling exhibited mild symptoms at 20 dpi (Figure 2a–c). The results showed that BxNMP1 plays a key role in the virulence of PWNs and can positively regulate their virulence. Moreover, the *P. thunbergii* plants infected by *BxNMP1*-silenced PWNs exhibited increased expression of PR genes, particularly *PR-1b*, in pine trees and decreased reproduction of PWNs in pine trees (Figure 2d and Figure S2). *PR1-b* is a marker gene of the plant SA pathway [43]. An increasing number of nematode effectors have been shown to suppress host immune responses. The cyst nematode effector Hs25A01 inhibits plant immune responses, increases nematode virulence, and affects host root morphology [48]. The southern RKN effector Minc03329 suppresses plant immunity and promotes plant infection [49]. Similarly, BxNMP1 can inhibit tobacco necrosis. Therefore, we speculated that the effector BxNMP1 regulates the virulence of PWNs by interfering with host immunity, especially the SA pathway, and PWN reproduction.

To explore the specific interaction mechanism between BxNMP1 and pine trees, we identified the target of BxNMP1 in *P. thunbergii*. The Y2H assay revealed that the target is *P. thunbergii* thaumatin-like protein-L2 (PtTLP-L2). Y2H and CoIP experiments confirmed the interaction between BxNMP1 and PtTLP-L2 (Figure 3). Studies have shown that thaumatin-like proteins have glucanase activity [39]. Interestingly, *P. thunbergii* β-1,3-glucanase (PtGLU) is also a candidate target of BxNMP1 (Table S2). However, Y2H experiments could not confirm the interaction between BxNMP1 and PtGLU (Figure 4a). Therefore, we speculate that PtTLP-L2 may act as a “communication bridge” between BxNMP1 and PtGLU. The results showed that PtTLP-L2 interacts with PtGLU in yeast (Figure 4a). A CoIP assay further verified this interaction (Figure 4b), confirming the interaction of two PR proteins of *P. thunbergii* for the first time. The three proteins are located in the nucleus and cytoplasm of plants, which also supports the possibility of spatial interactions among them (Figure 5). PR proteins are essential components of plant immunity [18,44,50]. However, the study of plant PR proteins has focused on the selection and breeding of resistant plants. For example, β-1,3-glucanase (PR2) and TLP (PR5) are commonly overexpressed to enhance plant disease resistance [22,24,25,51]. Although it has been shown that effectors can interact with PR proteins, no study has yet identified interactions among PR proteins [23,36,52]. Interestingly, TLPs are nonenzymatic antimicrobial proteins that render the cell membrane permeable, leading to osmotic imbalances and the death of pathogenic cells [53]. TLP families, as well as β-1,3 glucanase and members of the chitinase (PR3) family, act synergistically against pathogen cell membranes and cell walls [54,55]. This study showed that TLP and β-1,3 glucanase can interact with each other, so can these two proteins interact with chitinase (PR3), or can all the PR proteins in plants interact with each other? This hypothesis needs to be further verified with experiments.

Both TLP and β-1,3 glucanase respond positively to biotic or abiotic stresses in plants [19,24]. Similarly, we found that PtTLP-L2 and PtGLU respond to wound cutting or PWN inoculation. The upregulation of *PtTLP-L2* and *PtGLU* was more pronounced in *P. thunbergii* after nematode infection than after wound induction. In particular, at the later stages (10 d and 15 d), the relative expression of *PtTLP-L2* and *PtGLU* was more than 10,000-fold greater (Figure 6a,b). However, *BxNMP1* was highly expressed at the early stages (2.5 h, 6 h, 12 h, and 24 h) (Figure 1). The expression of *PtTLP-L2* and *PtGLU* peaked at 10 dpi when the pines began to show symptoms of PWD (Figure S3). At this time, the PWN is dominant in the interaction between PWNs and pine trees. Therefore, we speculate that the target’s relatively delayed response cannot prevent the onset of the disease. We subsequently inoculated the purified PtTLP-L2rec to establish the dominance of PtTLP-L2 at the beginning of the infection, and the results demonstrated a significant decrease in the morbidity of PWD. Based on these results, PtTLP-L2rec is expected to be used as an exogenous PWD control agent. Silencing *BxNMP1* upregulated the expression of *PtTLP-L2* and *PtGLU* in *P. thunbergii*, and the addition of PtTLP-L2rec induced the expression of these two targets in the host (Figure 7a). Therefore, BxNMP1 likely survives infection by inhibiting the expression of *PtTLP-L2* and *PtGLU* in the host. In nature, the successful invasion of pathogens may be attributed to the upregulation of key pathogenic genes in the early stages of infection, while the response of plant PR genes tends to lag.

SA is a signaling molecule that is crucial to the plant immune system. After plants are infected with living vegetative pathogens, salicylic acid (SA) synthesis in vivo increases dramatically, which is essential for activating plant resistance to pathogens [29]. Moreover, both β-1,3-glucanase (PR2) and thaumatin-like protein (PR5) are responsive genes of the salicylate pathway [43]. Numerous studies have demonstrated that pathogen effectors impact plant immunity by influencing the SA pathway in plants. *Meloidogyne incognita* chorismate mutase effectors localize to the cytoplasm and nucleus. They reduce the salicylic acid (SA) content of the host by half upon pathogenic infection and increase host susceptibility [56]. The effector SnTox3, secreted by *Parastagonospora nodorum*, can induce a potent necrotic response in wheat. It can also interact with wheat’s PR protein (TaPR-1) to activate the host SA pathway through preprotein release peptides, affecting plant immunity. Additionally, *PR1* serves as a marker response gene of the SA pathway [57]. The interaction between the t-SNARE domain effector of *Heterodera* spp. and resistance to cyst nematodes is influenced by the transcription of AtSHMT4 and AtNPR1, which affects the SA pathway in *Arabidopsis*, promoting nematode infection in Arabidopsis [36]. The primary synthesis pathway of SA in plants is the ICS pathway [29,58]. Glutamate is a component of the ICS pathway [59]. PAL is also closely related to plant defense responses [60]. The contents of SA, PAL, and glutamate synthase (GOGAT) increased in *P. thunbergii* seedlings inoculated with *BxNMP1*-silenced PWNs or with purified PtTLP-L2 protein (Figure 7b–d). These results indicate that PtTLP-L2 acts as an elicitor to activate the host immune response and SA pathway in plants. However, BxNMP1 can inhibit the expression of PtTLP-L2 and the plant ICS synthesis pathway to suppress the SA pathway in plants. When the expression of pathogen effectors is dominant, plant diseases will occur in nature.

In summary, we screened and identified a specific effector in PWNs that is localized in the dorsal gland and intestine of PWNs and is upregulated in the early stage of infection. Inoculation experiments revealed that BxNMP1 is a key factor in the virulence of PWNs. BxNMP1 directly interacts with PtTLP-L2 and indirectly interacts with PtGLU. This is the first time that interactions between two PR proteins in plants have been identified. Both targets belong to the plant SA pathway. BxNMP1 can inhibit the expression of two targets and the host pine SA pathway. Therefore, at the early stage of infection, the expression of *BxNMP1* is dominant, which inhibits the expression of targets and the plant SA pathway, resulting in the host being unable to resist PWNs. At the later stage of infection, the expression of *PtTLP* and *PtGLU* in pine trees was strongly upregulated, and pine trees wanted to exert their immune function to limit the development of PWNs. However, at this time, pine trees are beginning to show symptoms of PWD, and the strong immune response of the pine tree will only exacerbate the depletion of the pine and accelerate its own death. However, this study also revealed that the overexpression of PtTLP-L2 at the beginning of infection can reduce disease occurrence. Therefore, PtTLP-L2 can be used as a screening index for resistant pine trees. The addition of recombinant PtTLP-L2 protein can be used for the prevention and treatment of PWD. This study provides theoretical guidance and practical help for controlling PWD.

## 4. Materials and Methods

### 4.1. Biological Material

The pine wood nematode *Bursaphelenchus xylophilus* and the isolates LN16, ZJ07, CQ09, GX11, AMA3, YW4, JX06, OKD, and GD15 were obtained from Nanjing Forestry University Institute of Forestry Protection and cultured on PDA plates covered with *B. cinerea* mycelia at 25 °C for 7 days. The *B. xylophilus* were collected using the Baermann funnel technique. The tobacco used in this experiment was *N. benthamiana*. The culture conditions were 25 °C, 16 h of light and 8 h of darkness, and a relative humidity of 70–80%. The pine trees used in this experiment were three-year-old *P. thunbergii* seedlings sourced from Jurong Yaolingkou Forest Farm in Nanjing China.

### 4.2. Total RNA Extraction and cDNA Synthesis

Using TRIzol reagent, total RNA was extracted from PWNs (Yuanye Bio-Technology, Shanghai, China). Subsequently, the extracted RNA was reverse transcribed into cDNA using an Evo M-MLV RT kit for qPCR (Accurate Biology, Changsha, China).

### 4.3. BxNMP1 Gene Cloning, Plasmid Construction, and Real-Time Quantitative PCR

The *BxNMP1* gene was subsequently cloned from the cDNA of *B. xylophilus* (strain AMA3). Several recombinant plasmids of *BxNMP1* were constructed using homologous recombination (Vazyme, Nanjing, China). Subsequently, *Agrobacterium*-mediated transient expression experiments and protein induction, as well as yeast two-hybrid experiments, were performed. RT–qPCR was conducted using the SYBR Green Pro Taq HS Premix qPCR kit (Accurate Biology, Changsha, China) following the manufacturer’s instructions. All the primers used in this study are listed in Table S1.

### 4.4. Sequence Analysis and 3D Structure Modelling

The *BxNMP1* sequence species homology analysis experiment involved comparing the sequence with species in the NCBI database using BLASTP (https://blast.ncbi.nlm.nih.gov/Blast.cgi) (accessed on 23 December 2021). The signal peptide of *BxNMP1* was predicted using the SignalP v. 5.0 server (http://www.cbs.dtu.dk/services/SignalP/) (accessed on 23 December 2021). The domains of *BxNMP1* were analyzed using SMART (http://smart.embl-heidelberg.de/) (accessed on 23 December 2021) to explore the possible known domains, and we found that BxNMP1 contains no known domains. AlphaFold Colab was used to model the protein structure of *BxNMP1* [61]. The *BxNMP1* sequences of different PWN strains were compared using BioEdit version 7.0.9.

### 4.5. In Situ Hybridization

In situ hybridization was carried out using PWNs after infection with P. thunbergii for 12 h. Specific digoxigenin (DIG)-labeled probes were used to determine the localization of BxNMP1 in PWNs (Roche Diagnostics, Tokyo, Japan). The specific operational procedures can be found in the literature [62]. BxNMP1 localization in the PWNs was observed using a Zeiss microscope (Zeiss, Jena, Germany).

### 4.6. Transient Expression of BxNMP1 in N. benthamiana

The recombinant plasmid was inserted into *A. tumefaciens* via electroporation. Transgenic *A. tumefaciens* was injected into tobacco leaves. Total tobacco protein was extracted 36–48 h later, and the remaining tobacco was used to observe phenotypes.

### 4.7. Electrolyte Leakage Assay

An electrolyte leakage assay was used to quantify the extent of tobacco necrosis. A hole punch was used to extract 5 tobacco discs, which were then immersed in deionized water for 3 h to quantify the extent of electrolyte leakage. Electrolyte leakage assays were performed using the Five Easy Plus FE28 (METTLER TOLEDO, Columbus, OH, USA) system. All assays were repeated three times.

### 4.8. Total Protein Extraction and Western Blot Analysis

Tobacco leaves were ground into powder using liquid nitrogen, and then cell lysis buffer was added for Western blotting and IP to extract total protein from the tobacco plants. The extracted tobacco proteins were subjected to SDS–PAGE and membrane transfer experiments. The PVDF membranes were subjected to immunoblot analysis using antibodies conjugated to the corresponding antibodies. Equal protein loading was confirmed through Ponceau S staining of RuBisCO.

### 4.9. Detection of the Effect of BXNMP1 on B. xylophilus Pathogenicity

RNAi of BxNMP1 and inoculation assays were carried out as described previously [11,63]. Small interfering RNAs (siRNAs) corresponding to *BxNMP1* and the negative control *GFP* were synthesized using an in vitro Transcription T7 Kit (for siRNA Synthesis) (Takara, Kusatsu, Japan). The PWNs were immersed in the siRNA solution for 48 h, and the reaction was stopped by washing three times with double-distilled water. RT–qPCR was used to determine the interference efficiency of *BxNMP1*.

### 4.10. Y2H and Co-IP Assays

*BxNMP1* and *PtGLU* (without a signal peptide) were cloned and inserted into the pGBKT7 bait vector and then transformed into the yeast strain Y2H Gold. The cDNA library of *P. thunbergii* inoculated with *B. xylophilus* was screened following Clontech protocols. Subsequently, the potential targets were cloned and inserted into the pGADT7 vector to conduct a Y2H assay. The complete alignment of the potential interactors *PtTLP-L2* was then cloned and inserted into pGADT7 using the Clone Express II One Step Cloning Kit (Vazyme, Nanjing, China). The interaction between pGBKT7:*PtGLU* and pGADT7:*PtTLP-L2* in yeast was verified via the same methods. For the Co-IP assay, *BxNMP1* and *PtGLU* were cloned and inserted into pBINRFP, and *PtTLP-L2* was cloned and inserted into pBINGFP. All constructs were introduced into *A. tumefaciens* GV3101 via electroporation. Cell suspensions (pBINRFP:*BxNMP1* and pBINGFP:*PtTLP-L2*) and mixed bacterial solutions (pBINRFP:*PtGLU* and pBINGFP:*PtTLP-L2*) were infiltrated into *N. benthamiana*. At 48 h after infiltration, the proteins were extracted, and CoIP assays were performed as previously described [64].

### 4.11. Confocal Microscopy

The transgenic *A. tumefaciens* was subsequently injected into tobacco plants. After 36–48 h, tobacco leaves were extracted using a hole punch to create a temporary slide. The fluorescence localization was then observed under a Zeiss microscope.

### 4.12. PtTLP-L2 and PtGLU Gene Cloning, Plasmid Construction, and Real-Time RT–qPCR Analyses

The coding sequences of *PtTLP-L2* and *PtGLU* were amplified from *P. thunbergii* cDNA using specific primers. Gene cloning and real-time RT–qPCR analyses were performed as described previously.

### 4.13. Expression and Purification of the Recombinant PtTLP-L2 Protein

*PtTLP-L2* (without a signal peptide) was cloned and inserted into the prokaryotic expression vector pET32a, while the pET32a empty vector (EV) was utilized as a negative control. The recombinant plasmid and the empty vector were subsequently transformed into the *Escherichia coli* strain BL21 via heat shock, which was subsequently validated via PCR and subsequently sequenced as previously described. In vitro expression of PtTLP-L2 was induced at 25 °C for 12 h with 1 mM IPTG, as described previously [65].

### 4.14. Detection of Salicylic Acid and Related Enzyme Activities

To analyze the role of BxNMP1 in regulating host immunity, the levels of salicylic acid (SA) and the activities of phenylalanine ammonia lyase (PAL) and glutamate synthase (GOGAT) were determined in the stems of *P. thunbergii* plants inoculated with PWNs or purified PtTLP-L2 protein 12 h later. The stems were frozen in liquid nitrogen and ground into a fine powder using a mortar and pestle. The SA content was determined following the instructions of the Plant Salicylic Acid ELISA Kit (Mosakbio, Wuhan, China). The activity assays of PAL and GOGAT were conducted in accordance with their respective instructions (Molfarming, Nanjing, China; Solarbio, Beijing, China).

## Data Availability

The data that support the findings of this study are available from the corresponding author upon reasonable request.

## Supplementary Materials

The supporting information can be downloaded at: https://www.mdpi.com/article/10.3390/ijms25137452/s1.
