# Supplementary material for: The Bursaphelenchus xylophilus Effector BxNMP1 Targets PtTLP-L2 to Mediate PtGLU Promoting Parasitism and Virulence in Pinus thunbergii
Source: Int J Mol Sci. 2024 Jul 7;25(13):7452. doi: 10.3390/ijms25137452 (PMC11242139; doi:10.3390/ijms25137452)
Supplement: Supplementary file 1 [file ijms-25-07452-s001.zip › ijms-3027603-supplementary.pdf]

Supporting information

Table S1 List of primers used in this study

Primer name	Forward primer sequence (5'-3')	Reverse primer sequence (5'-3')	Purpose
BxNMP1	ATAGCCGGTACCCCCGGGATGCGTCTTTTGCTGGTTGC	GGAGGAGGCCATCCCGGGCAATTGAACCTGAAAGTATCC	Contrust the plasmid of pBINRFP: BxNMP1
BxNMP1^{-SP}	ATAGCCGGTACCCCCGGGATGGATTGTGCGGAAGGACCATG	GGAGGAGGCCATCCCGGGCAATTGAACCTGAAAGTATCC	Contrust the plasmid of pBINRFP: BxNMP1^{-SP}
BxNMP1:BD	GCCATGGAGGCCGAATTCATGGATTGTGCGGAAGGACCATG	ACGGATCCCCGGGAATTCCAATTGAACCTGAAAGTATCC	Contrust the plasmid of pGBKT7: BxNMP1^{-SP}
BxNMP1:AD	GCCATGGAGGCCAGTGAATTCATGGATTGTGCGGAAGGACCATG	ATGCCCACCCGGGTGGAATTCCAATTGAACCTGAAAGTATCC	Contrust the plasmid of pGADT7: BxNMP1^{-SP}
Actin	GCAACACGGAGTTCGTTGTA	GTATCGTCACCAACTGGGAT	RT-qPCR endogenous control for *Bursaphelenchus xylophilus*
RT-qPCR BxNMP1	GAAGGACCATGCCAGAGCTT	CGGCTTCGAGCAGTGGTATT	RT-qPCR for *BxNMP1* expression levels in *B. xylophilus*
Sense BxNMP1	TAATACGACTCACTATAGGGATGCGTCTTTTGCTGGTTGC	TGCACAACCAGCTGCTCCA	In situ hybridization
Antisense BxNMP1	ATGCGTCTTTTGCTGGTTGC	TAATACGACTCACTATAGGGCAATTGAACCTGAAAGTATCC	In situ hybridization
BxNMP1 oligo-1/2	GATCACTAATACGACTCACTATAGG<br>GGCAATGGAGGATACTTTCAGGTTC ATT	AATGAACCTGAAAGTATCCTCCATT<br>GCCCCTATAGTGAGTCGTATTAGTG ATC	Amplification for synthesizing BxSCD3siRNA and GFP siRNA to RNAi
BxNMP1 oligo-3/4	AAGCAATGGAGGATACTTTCAGGTT<br>CACCCTATAGTGAGTCGTATTAGTG ATC	GATCACTAATACGACTCACTATAGG<br>GTGAACCTGAAAGTATCCTCCATTG CTT	
GFP oligo-1/2	GATCACTAATACGACTCACTATAGGGATGAGTAAAGGAGAAGAACTTTTCTT	TTCTTTTCAAGAAGAGGAAATGAGTAGGGATATCACTCAGCATAATCACTAG	
GFP oligo-3/4	AAATGAGTAAAGGAGAAGAACTTTTCCCCTATAGTGAGTCGTATTAGTGATC	CTAGTGATTATGCTGAGTGATATCCCCTTTTCAAGAAGAGGAAATGAGTAAA	
PtEF1α	AGATTGGAAATGGTTATGCCCCTGT	CCAGAACGCCTGTCAACCTTGGT	RT-qPCR endogenous control for *Pinus thunbergii*
PtPR-1b	TGCCCCTTCAGGTAAATCGT	GCGGGTCGTAGTTGCAGATAA	RT-qPCR for *PR-1b* expression levels in *P. thunbergii*
PtPR-2 (beta-1, 3-glucanase)	CGACAACATTCGCCCCTTCT	CTGCAGCGCGGTTTGAATAT	RT-qPCR for *PtPR-2* (beta-1, 3-glucanase) expression levels in *P. thunbergii*
PtPR-3-II (class IV chitinase)	CCATCGAAGCCCAGGTAATTT	AGCCGGGAAGCAATATTATGGT	RT-qPCR for *PR-3-II* (class IV chitinase) expression levels in *P. thunbergii*
PtPR-5 (thaumatin like protein)	GAACCAGTGCCCATACACAGTCT	CCTGCGGCAACGTTAAAAGTC	RT-qPCR for *PtPR-5* (thaumatin like protein) expression levels in *P. thunbergii*
PtPR-6 (type II	TGCTGGCGGCATCTATTT	TAACACCTGCGCAAATGCA	RT-qPCR for *PR-6*

proteinase inhibitor family protein)	TA		expression levels in *P. thunbergii*
PtPR-9 (peroxidase)	ACACCACCGTGCTGGACATT	GTGCGGGAGTCGGTGTAGAG	RT-qPCR for *PtPR-9* (peroxidase) expression levels in *P. thunbergii*
PtTLP-L2	CCCTCTTTCTGCAACTCCGT	GTGCAGCAGTATTGAGGGGT	RT-qPCR for *PtTLP-L2* expression levels in *P. thunbergii*
PtTLP-L2:AD	GCCATGGAGGCCAGTGAATTCATGGCGACGTTCACAGTGAGGAA	ATGCCCACCCGGGTGGAATTCACCGCAGAAGACGACGTTGT	Contrust the plasmid of pGADT7: PtTLP-L2^{-SP}
PtTLP-L2:32a	AAGGCCATGGCTGATATCATGGCGACGTTCACAGTGAGGAA	GAATTCGGATCCGATATCACCGCAGAAGACGACGTTGT	Contrust the plasmid of pET32a: PtTLP-L2^{-SP}
PtTLP-L2:RFP	ATAGCCGGTACCCCCGGGATGGCGACGTTCACAGTGAGGAA	GGAGGAGGCCATCCCGGGACCGCAGAAGACGACGTTGT	Contrust the plasmid of pBINRFP: PtTLP-L2^{-SP}
PtGLU	TTTCGATCCAGACGGTGGTG	ATACGTACTGCGTGCTAGGC	RT-qPCR for *PtGLU* expression levels in *P. thunbergii*
PtGLU:AD	GCCATGGAGGCCAGTGAATTCATGGGATAAAATAGGAGTGAACTA	ATGCCCACCCGGGTGGAATTCTGGAGAAAAGTTGACAGAGT	Contrust the plasmid of pGADT7: PtGLU^{-SP}
PtGLU:BD	GCCATGGAGGCCGAATTCATGGGATAAAATAGGAGTGAACTA	ACGGATCCCCGGGAATTCTGGAGAAAAGTTGACAGAGT	Contrust the plasmid of pGBKT7: PtGLU^{-SP}
PtGLU:RFP	ATAGCCGGTACCCCCGGGATGGGATAAAATAGGAGTGAACTA	GGAGGAGGCCATCCCGGGTGGAGAAAAGTTGACAGAGT	Contrust the plasmid of pBINRFP: PtGLU^{-SP}

Table S2 BxNMP1 potential targets for the Y2H assay

BxNMP1 potential targets for the Y2H assay	
**structural domain**	**Captured times**
Thaumatin-like protein	12
Cyclophilin protein	8
Elongation factor	5
β-1,3-glucanase	5
Chitinase	4
1-aminocyclopropane-1-carboxylate oxidase 1	3
isopentenyl diphosphate delta-isomerase	3
Aquaporin	1
POX1	1

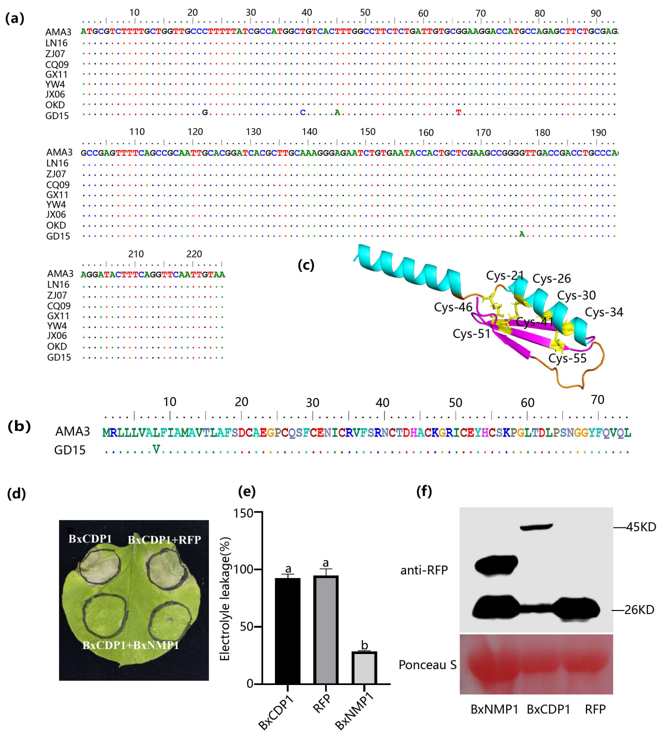

Figure S1. Identification of the *Bursaphelenchus xylophilus* candidate effector BxNMP1. (a) Sequence polymorphism analysis of *B. xylophilus* strains of different origins and virulence. (b) Amino acid sequence analysis of the highly virulent strain AMA3 and the weakly virulent strain GD15. (c) Protein structure model of BXNMP1. (d) Co-expression of BxNMP1 (with signal peptide) suppressed BxCDP1-triggered hypersensitive cell death in *Nicotiana benthamiana*. Pictures were taken 5 days post infiltration. The experiments were repeated at least three times with similar results. (e) Quantification of suppression of cell death by measuring electrolyte leakage in N. benthamiana. RFP, red fluorescent protein. The data shown are combined from three independent experiments. Values represent the mean ± SD of three independent biological samples. Different letters over error bars indicate statistically significant differences using Duncan's multiple range test ($p < 0.05$). (f) Immunoblot analysis of proteins from *N. benthamiana* leaves transiently expressing target proteins. Protein loading is indicated by Ponceau S staining of RuBisCO.

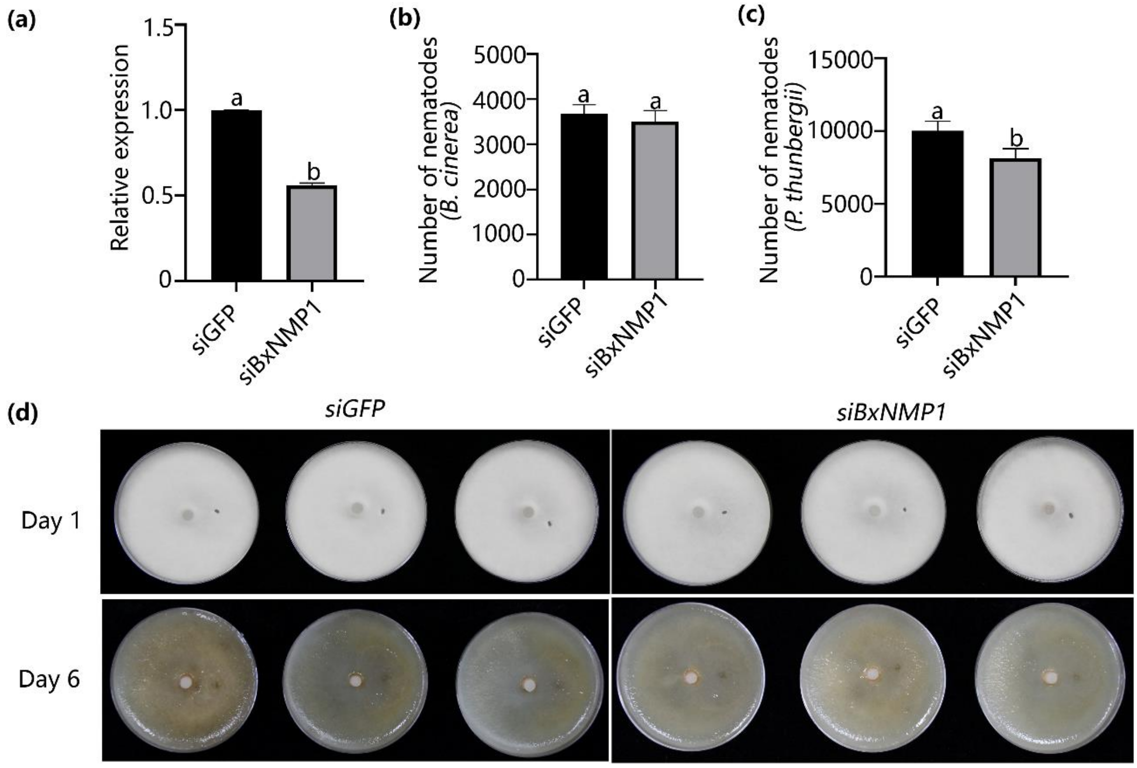

Figure S2. The effect of *BxNMP1* silencing on the reproduction and feeding rate of *Bursaphelenchus xylophilus*. (a) The silencing efficiency of *BxNMP1* in *B. xylophilus*. (b) The number of nematodes in *Pinus thunbergii* inoculated with dsRNA-treated nematodes. In total, 2000 nematodes were inoculated per pine tree and sampled at 20 days postinoculation. (c) The number of nematodes on *Botrytis cinerea* over 6 days. Each dish of *B. cinerea* was inoculated with 100 nematodes. Values represent the mean ± SD of three independent biological samples. Different letters over error bars indicate statistically significant differences using Duncan's multiple range test ($p < 0.05$). (d) The propagating quantity of *B. xylophilus* cultured on *B. cinerea*.

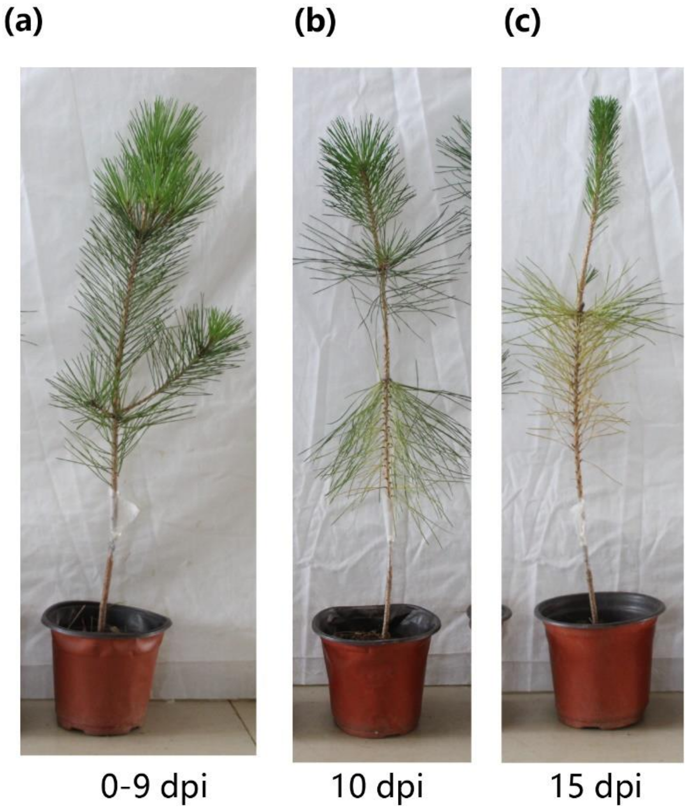

Figure S3 Symptoms of pine wilt disease at different infection stages for reverse transcription–quantitative PCR analysing. *Bursaphelenchus xylophilus* was inoculated into *Pinus thunbergii* seedlings. The *B. xylophilus* were collected by Belman funnel, and the total RNA of *P. thunbergii* was extracted by removing the 1cm pine stem segment near the inoculation point. (a) pine trees inoculated with *B. xylophilus* showed no symptoms of pine wilt disease 9 days postinoculation (dpi). (b) At the 10 dpi, a few pine needles began to turn yellow. (c) most pine needles inoculated with *B. xylophilus* started to turn yellow at the 15 dpi with Bursaphelenchus xylophilus.

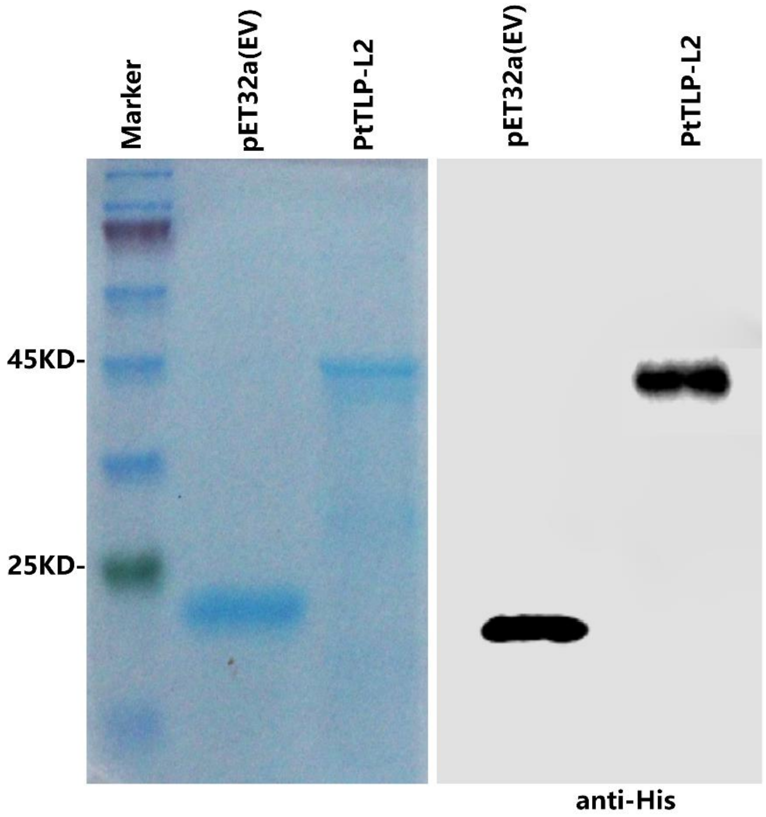

Figure S4. Sodium dodecyl sulphate-polyacrylamide gel electrophoresis (SDS-PAGE) and western blot verification of the PtTLP-L2 protein.
